# Thermometry of stored molecular ion beams

**DOI:** 10.1038/s41598-022-26797-5

**Published:** 2022-12-29

**Authors:** Abhishek Shahi, Deepak Sharma, Sunil Kumar, Saurabh Mishra, Igor Rahinov, Oded Heber, Daniel Zajfman

**Affiliations:** 1 Department of Particle Physics and Astrophysics, Weizmann Institute of Science, 7610001 Rehovot, Israel; 2 Department of Natural Sciences, The Open University of Israel, 4310701 Ra’anana, Israel; 3 Present Address: Saha Institute of Nuclear Physics, 1/AF, Bidhannagar, Calcutta, 700064 India

**Keywords:** Physics, Atomic and molecular physics, Chemical physics, Techniques and instrumentation

## Abstract

The radiative cooling of a stored, initially rotationally hot OH$^{-}$ ion beam is probed by photodetachment using an electrostatic ion beam trap combined with an in-trap velocity map imaging spectrometer, providing direct measurement of the time-dependent rotational population. The rotational temperatures are estimated from photodetached electron spectra as a function of time using a Boltzmann distribution model and further verified by a rate law model using known Einstein coefficients. We demonstrate that during the entire cooling time, the rotational population can be well described by a Boltzmann distribution.

## Introduction

Information on the internal energy states of molecular ions is a prerequisite for many experiments, as well as for comparison to theoretical calculations and astrophysical observations^1^. Such comparison can be challenging, since experimentally molecular ions are usually produced by a violent act that leaves them in a superposition of excited states, which are dependent on the production process, the environment as well as the time scale. Given that the rates of many chemical reactions are, in general, highly dependent on temperature, it becomes important to be able to devise experiments where the temperature, or the actual internal energy distribution of the molecular ion reactant could be probed. In addition, time-dependent measurement of the state population would allow for a better understanding of the internal molecular dynamics, as well as a direct comparison with (or measurement of) state selective lifetimes or Einstein coefficients. Moreover, while most environments where molecular ions are involved in reactions (plasma, molecular clouds and others) are usually defined by an equilibrium temperature, it is not always clear whether in an experiment the population of the molecular ion rotational states faithfully replicates the expected Boltzmann distribution at thermal equilibrium.

Various methods have been developed to produce cold (i.e. ground state) molecular ions^2^. Among them, a few popular methods are the supersonic expansion techniques^3, 4^ and the ion trapping methods where cooling is achieved through collision with a buffer gas^5^. At a different scale of size and beam energy, the heavy ion storage ring^6^, and later on, the electrostatic ion beam trap (EIBT)^7–10^ techniques have demonstrated their advantages for studying infrared active molecular ions. In such a setting, a beam of hot and fast (keV to MeV) molecular ions is injected into a storage ring or an EIBT, and relaxation of the internal degrees of freedom occurs through spontaneous emission of the electronic, vibrational, and rotational states. The asymptotic internal temperature of the molecular ions is limited by the blackbody radiation emitted by the wall of the ring or the trap. Recently, a new generation of cryogenically cooled storage rings^11–13^ and EIBT^14^ has reached a temperature of the order of 10–20 K, allowing for almost complete relaxation of the internal degrees of freedom. While typical radiative (allowed) electronic and vibrational relaxation times are of the order of ns to several seconds, the rotational cooling can extend to several minutes, and even hours. Although the asymptotic equilibrium temperature is known (regulated by the blackbody radiation), these techniques cannot be easily used to probe and follow the internal dynamics (mostly the population of rotational states) over a large range of temperatures.

A few experiments have successfully measured the rotational state population of stored molecular anions in cryogenic storage rings using near-threshold photodetachment^15, 16^. In such experiments, a tunable laser is merged with the stored beam at different times and various wavelengths. By measuring the photodetachment cross-section at different wavelengths and times, it is possible to extract the population changes of the rotational states. While such experiments can precisely measure the rotational population at relatively low temperatures (tens of K), they are more difficult to apply at higher temperatures when many rotational states are still populated^15, 16^.

In the following, we present a new method using OH$^{-}$ to measure the rotational temperature of stored anions. Although the technique is not as precise as the threshold photodetachment method used in previous experiments^15, 16^, it is more general, works over a larger range of temperatures, and provides a direct estimation of the rotational population during the entire storage time.

The photodetachment of OH$^{-}$ has been measured in a number of experiments^15–19, 19–21, 21–23^ and its structure, energetic and potential energy surfaces have been calculated in several theoretical works^24^, and references therein. OH$^{-}$ ion is one of the most suitable candidates to explore photodetachment thermometry because the ground states of the anion and the neutral specie are dipole-bound states, the photodissociative potentials lie well above the photodetachment potentials^24, 25^, and the rotational lines spacings are large, a feature particularly interesting for high resolution photoelectron spectra.

**Figure 1:**
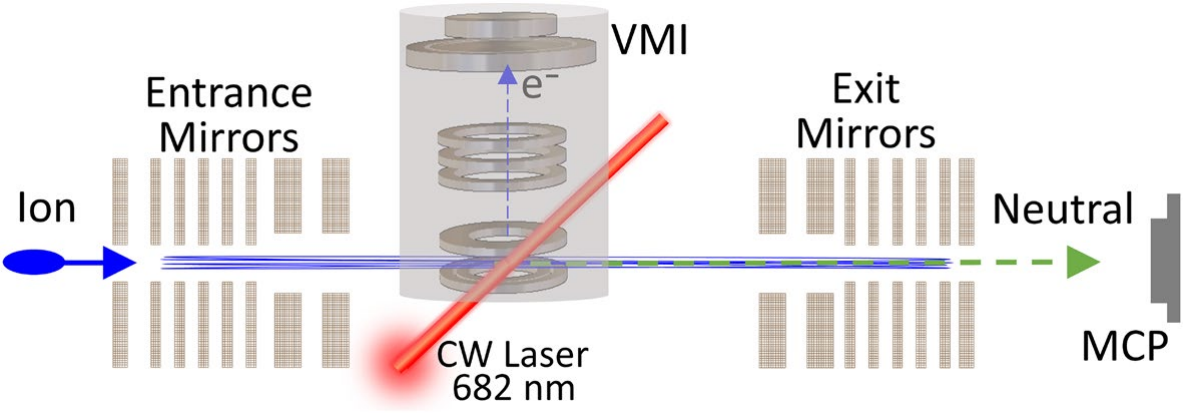
Schematic of the experimental setup. A bunch of OH$^{-}$ is injected in the EIBT when the entrance voltages of the left mirror electrodes are low. After the voltages are raised, the beam is trapped between the two mirrors, and the anions are photodetached by a CW laser. The electrons kinetic energy is analyzed using a VMI spectrometer.

## Results and experimental description

**Figure 2:**
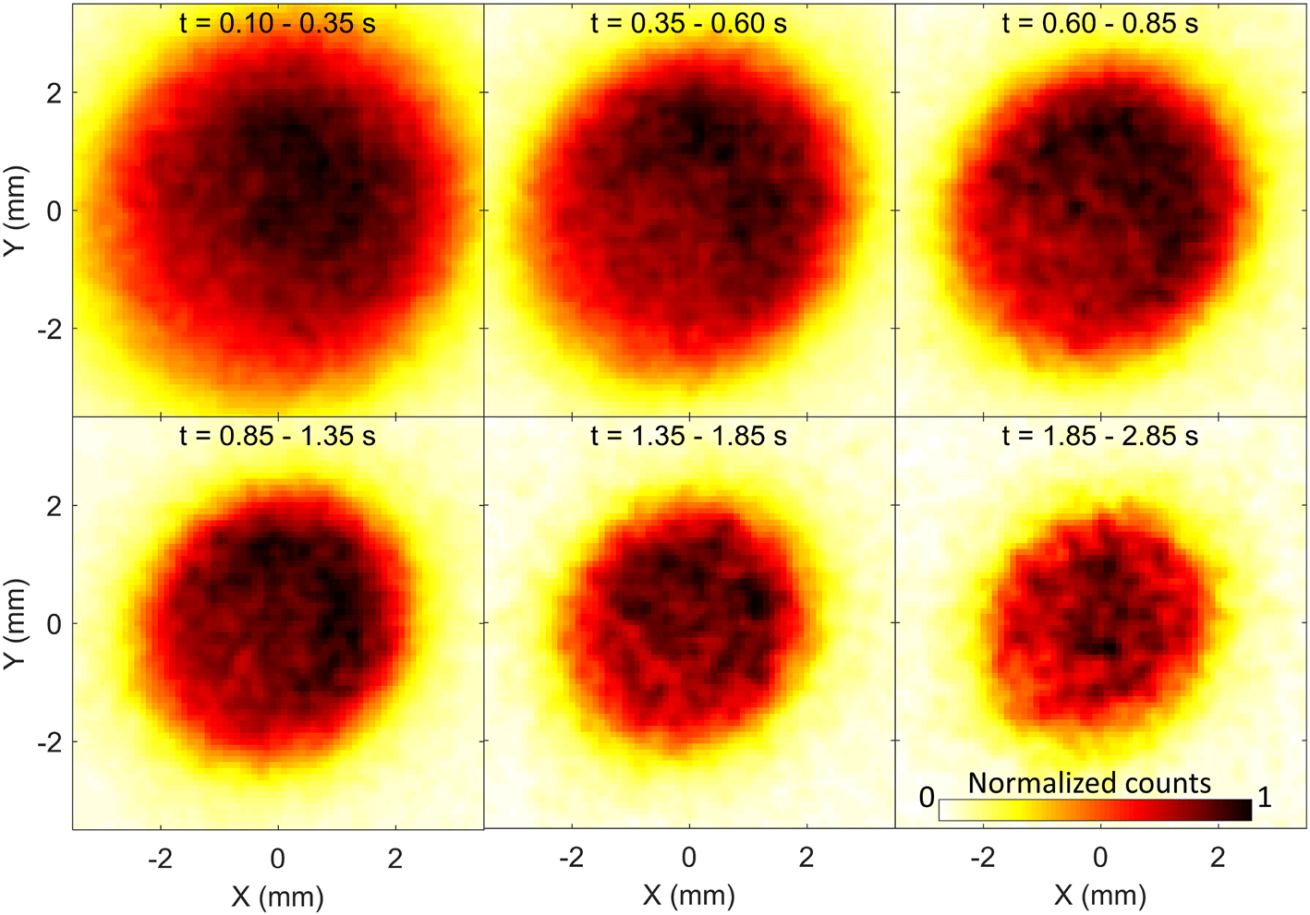
VMI of the photodetached electrons as a function of trapping times.

In the present experiment, a beam of OH$^{-}$ (electron affinity = 1.8277 eV^20^) is produced in a Cs sputter ion source, accelerated to an energy of 4.2 keV and injected into an EIBT^7^. A schematic of the experimental setup is shown in Fig. 1. A velocity map imaging (VMI) set-up is installed in the field-free region, inside the EIBT to record the photoelectron spectra^26–28^. Once injected in the EIBT (where the pressure is about P = 2–3 $\times$ 10$^{-10}$ Torr), the stored particles oscillate between the two mirrors, with a lifetime of 435 ms. The measurement is stopped after 3 s, the residual stored beam is released by lowering the mirrors voltages, followed by a new injection for the next round of measurement.We estimate the ion number density by the DC ion current before the injection to the trap to be about 5 $\times$ 10$^{3}$ ions/cm$^{3}$ at the time zero and about 100 times less after 3 s (with or without the laser). The anions are crossed by a continuous wave (CW) laser beam with a photon energy of $h\nu$ = 1.818 eV, at the middle of the VMI setup. The time of flight and position of the photodetached electrons are recorded by the VMI microchannel plate (MCP) detector, in coincidence with the resulting neutral OH, as detected by the MCP located outside the EIBT (see Fig 1). Each detector has an efficiency of about 50%. Coincidence efficiency is a multiplication of the detector efficiency divided by two (fragments counts only on one side of the trap), resulting in coincidence efficiency of about 12%. The VMI raw data for different trapping time ranges are shown in Fig. 2. The analysis starts, for each injection, after 100 ms of storage time to avoid complications due to unstable ion beam trajectories in the EIBT. As can be seen in Fig. 2, the size of the electron spot on the detector, which is directly related to the electron kinetic energy, is decreasing over time, suggesting that the initially hot OH$^{-}$ anions are cooling.

Using well-known VMI analysis techniques, these images are first centralized, circularized, and then inverse Abel transformed^29^ to obtain the photodetached electron kinetic energy as a function of the storage time. A preliminary experiment performed under the same condition with O$^{-}$ (electron detachment energy = 1.461 eV^30^) allows for a precise calibration of the VMI setup (Fig. S1 of supporting information, SI-1). Background was subtracted by measuring VMI spectra without a laser (Fig. S2 of SI-2).

**Figure 3:**
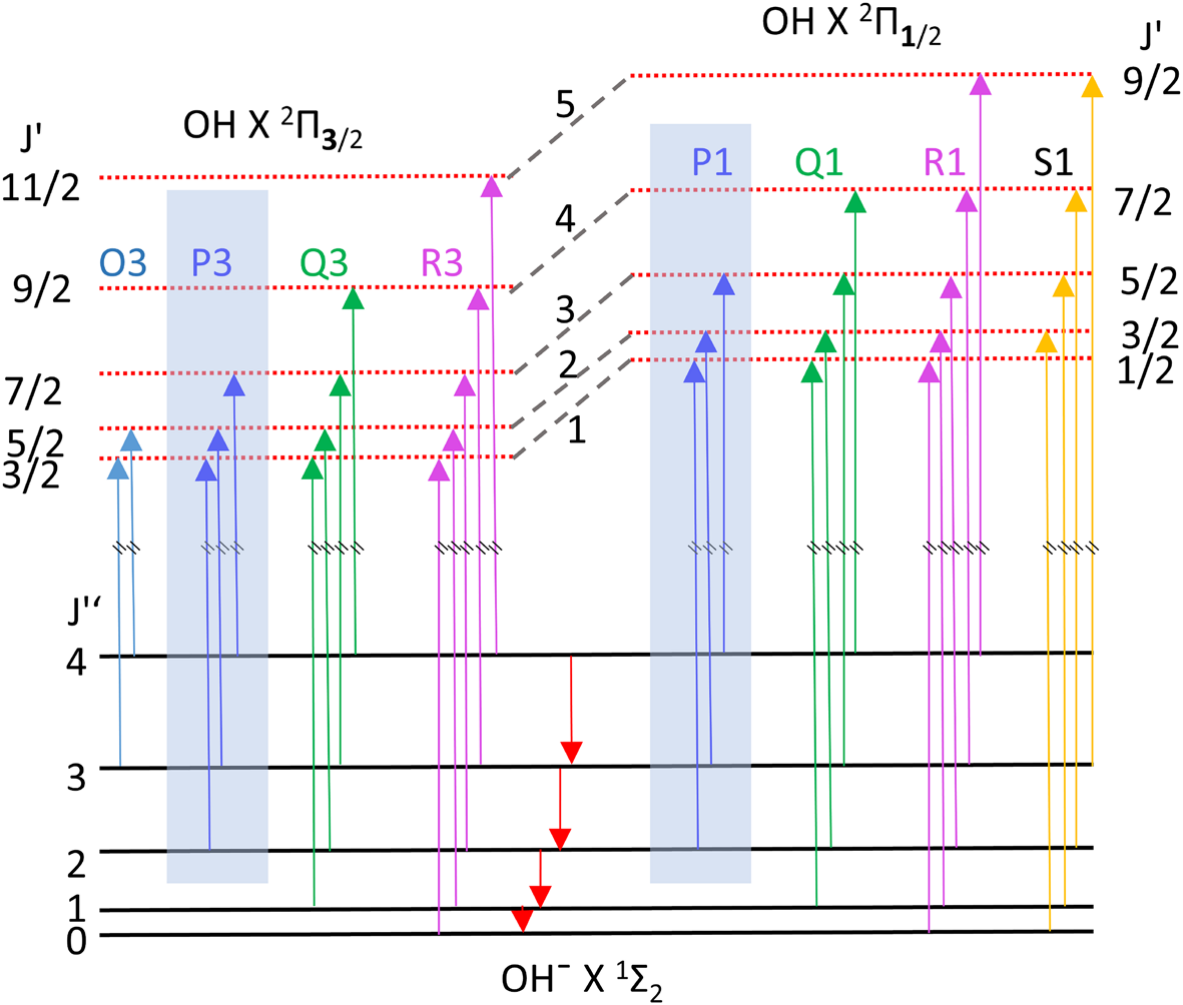
Jablonski diagram representing anion to neutral photon transitions (for the few lowest rotational levels as an example). Downward pointing red arrows are spontaneous emission.

**Figure 4:**
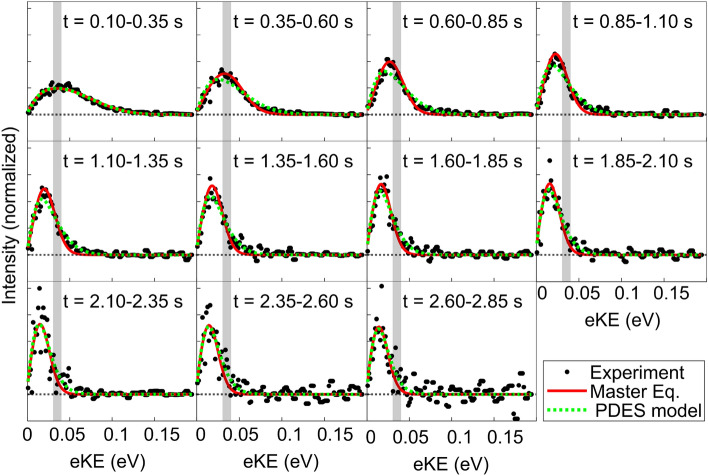
Experimental photoelectron spectra (black circles) for various storage time windows starting from 0.1 to 3 s. The green dotted lines are the fitted photodetachment electron spectrum model distributions. The solutions of the Master equation (Eq. 3) are shown by the red solid lines. The shaded vertical rectangles indicate the initial location of the peak for the first time window (upper left panel). Due to the beam lifetime the number of ions left in the trap is decreased, therefore resulting in a lower signal to noise, as reflected in the later trapping time windows.

The resulting photoelectron kinetic energy spectra are shown in Fig. 4 (black circles) for various storage time windows. It is immediately clear from the data that the electron kinetic energy distribution is both narrowing and moving to lower energies with time, a clear sign of the cooling of the internal degrees of freedom of OH$^{-}$. For convenience, a vertical shaded area is plotted, indicating the position of the distribution peak during the first time window (0.1–0.35 s). The negative data points observed at the latter storage time are the result of the background subtraction and the limited statistics available at these longer storage times.

## Discussion

In order to extract the time-dependent temperature of the stored OH$^{-}$, we assume that the electron kinetic energy distributions, for each time window, are the result of a thermal Boltzmann distribution of the OH$^{-}$rotational states. To test this hypothesis, we use the well-known spectroscopic structural constants of OH and OH$^{-}$^18, 31^. After electron detachment, the resulting neutral OH exhibits two states $X^2 \Pi _{3/2}$ and $X^2 \Pi _{1/2}$ due to spin-orbit coupling^17^. The corresponding transitions are labeled as ‘3’ and ‘1’, respectively. Among all possible P3, P1, Q3, Q1, R3 and R1 transitions, the selected photon energy allows to probe only P3 and P1 transitions. An energy diagram representing anion to neutral transitions is shown in Fig. 3. Based on previous experiments^17, 30^, it is known that the initial population of vibrational excited states of OH$^{-}$ is negligible. Also, using the calculated dipole moment of OH$^{-}$, the estimated lifetime of the v = 1 state is of the order of 7 ms^25^, which is much smaller than the time delay between the beam injection and the start of the measurement (100 ms). Using j-dependent photoelectron detachment model^15, 18^, the threshold transition intensities ${\hat{I}}_{j}$ of P3 and P1 transitions are calculated where *j* is OH$^{-}$ rotational quantum numbers (directly taken from references^15, 20^).

Assuming a Boltzmann distribution $C(j,T)\propto (2j+1) e^{-\frac{E_{j}}{kT}}$ where *T* is the molecular rotational temperature and $E_{j}$ and *C*(*j*, *T*) are the rotational energy and the relative population of the *j* level of OH$^{-}$, the total intensities, $I_{j}$, of P3 and P1 transitions can be estimated as^15, 16^

1 $$\begin{aligned} I_{j} = N {\hat{I}}_{j} (h\nu - h\nu _j)^a *C(j,T) \end{aligned}$$

where $h\nu$, $h\nu _j$ and *a* are the photon energy, the transition energies for P3 and P1 transitions and the Wigner factor, respectively. In this study, *a* is set to 0.2^15^, and *N* is a normalization constant. Although the partition function is temperature dependent, it has only a constant value for a given temperature (implicitly included in the normalization factor). Since we use only relative populations it does not affect the extracted temperature when the area under the distribution is normalized to unity (as was done in this work).

In order to fit the experimental data shown in Fig. 4, the distribution of rotational level energies are converted to a distribution of electron kinetic energies by energy conservation:

2 $$\begin{aligned} eKE_{(j)} = h\nu - \overline{h\nu }_{(j)} \end{aligned}$$

where $\overline{h\nu }_{(j)}$ are the energies for P3 and P1 transitions written in terms of OH$^{-}$ rotational levels *j*. $\overline{h\nu }_{(j)}$ is calculated using the electron affinity and the rotational level energies of OH$^{-}$ and $X^2 \Pi _{3/2}$ and $X^2 \Pi _{1/2}$ states of OH, including selection rules.

Using Eqs. (1) and  (2), the photodetached electron spectrum (PDES) shown in Fig. 4 is fitted with *T* as a free parameter (and *N* as a normalization factor), and shown as a green dotted line. As can be seen, the assumption that the electron kinetic energy is the result of a Boltzmann distribution for the rotational state of OH$^{-}$ yields a reasonable fit to the experimental data, for most of the storage time windows, although there are visible discrepancies, especially during the first second of storage.

**Figure 5:**
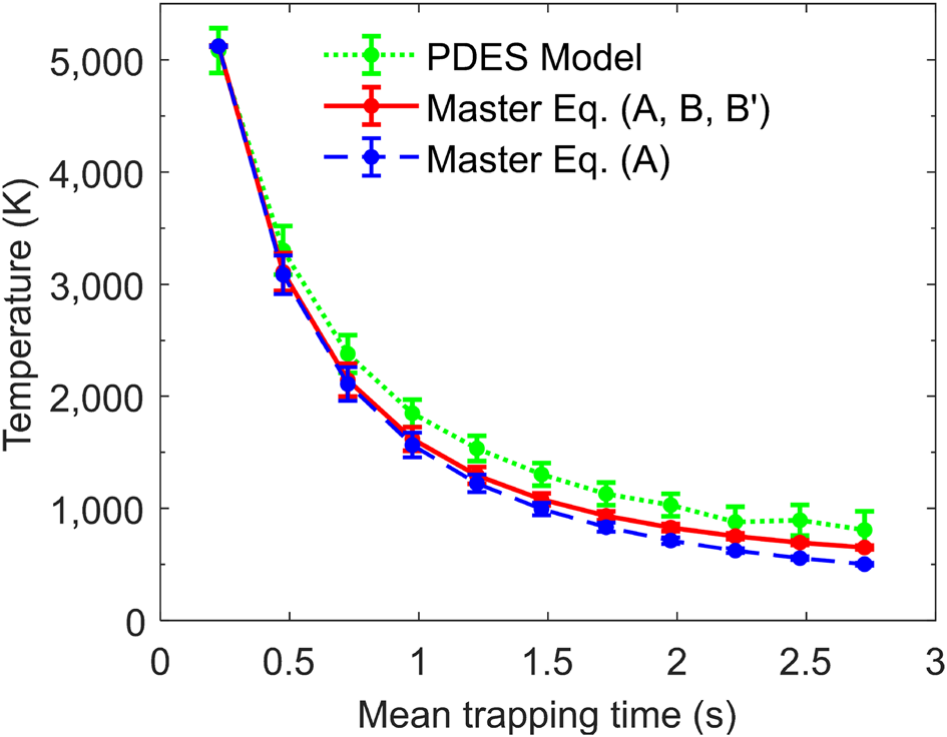
The green dots are experimentally measured time-dependent temperatures obtained assuming a Boltzmann distribution for the rotational state of OH$^{-}$. Also shown are the calculated time-dependent temperatures from the Master equation under two different assumptions: without blackbody radiation (blue dots) and with blackbody radiation (red dots). The error bars of the temperature are estimated at 95% confidence level when fitting to PDES model. The lines are drawn to guide the eyes.

The resulting fitted temperatures *T* are plotted as a function of time in Fig. 5, shown by green dots. These results demonstrate the rapid cooling of the rotational states of the stored OH$^{-}$ over the 3 s of storage, starting from a temperature of about 5100 K for the initial time window of 0.1–0.35 s down to about 800 K after 2.75 s of storage. Note that the fitted initial temperature, 5100 ± 200 K in this study is in consistence with the estimated initial temperature of 6000 ± 2000 K by Meyer et al.^15^, where the molecular ions were produced in a similar ion source.

To support the analysis of the data shown in Fig. 4 we compare the results shown in Fig.5 to the expected cooling rate obtained from the known Einstein coefficients of OH$^{-}$. To perform such an analysis, the time-dependent population $P_j$ of OH$^{-}$ rotational level *j* is calculated using the following Master equation:

3 $$\begin{aligned} \frac{dP_j}{dt} = B_{j}*P_{j-1} - (A_{j}+B_{j+1}+B'_{j})*P_{j} + (A_{j+1}+ B'_{j+1})*P_{j+1} \end{aligned}$$

where $A_j$, $B'_j$ and $B_j$ are the Einstein coefficients in the units of $s^{-1}$ between *j* and $j-1$ rotational levels, namely spontaneous emission, stimulated emission and photon absorption, respectively. The later two processes take place due to the thermal blackbody radiation from the EIBT environment. Given that the experiment is performed at room temperature, the blackbody spectrum is calculated at 300 K.

In order to solve Eq. (3), the initial rotational state population (temperature) for the first time window [0.1–0.35] s is assumed to be identical to the one extracted in the analysis above. Up to 50 rotational states are taken into account, yielding a set of 50 coupled differential equations. The Einstein coefficients for the OH$^{-}$ rotational transitions are evaluated based on the known values of transition energy, internuclear dipole moment and blackbody photon number density^22, 32, 33^.

The solution of the differential equations (3) yields the time-dependent population of OH$^{-}$, which can then be used to create “synthetic” electron energy spectra, to be compared to the experimental data shown in Fig. 4 as solid red lines. A better agreement than the one assuming a simple Boltzmann distribution (green dotted lines) is obtained between this model and the experimental data. Note that except for the initial population, there are no fitting parameters in this model, supporting the theoretical values for the Einstein coefficients and the spectroscopic constants^22, 32, 33^, within the experimental resolution.

To provide an overall comparison between the photoelectron spectrum model and the Master equation (Eq. 3), a time-dependent temperature can be extracted from the solution of the differential equations by fitting them to a Boltzmann distribution. The results are shown in Fig. 5 as red and blue dots. The agreement with the analysis performed under the assumption that the data can be represented by a Boltzmann distribution at all time (green dots) is very reasonable, demonstrating the validity of such an assumption. Also shown in Fig. 5 (red dots) is the solution of Eq. (3) with the effect of blackbody radiation (i.e., including all Einstein coefficients $A_j$, $B_j$ and $B'_j$). As expected, given the relatively high temperature of the molecular ions at the beginning of the measurement (5100 K) and at the end (800 K), the influence of spontaneous emission is most significant. Nevertheless, as the rotational cooling takes place, and the temperature decreases, the effect of the blackbody radiation becomes visible, and the calculated cooling rate decreases. The Master equation was checked by calculating that, for much longer storage time, the rotational population indeed reaches the expected value for 300 K when blackbody radiation is included, and 0 K (i.e., only the j = 0 state being populated) when $B_j=B'_j=0$.

## Conclusion

In conclusion, the relaxation behavior of rotationally hot OH$^{-}$ has been measured using a combined method of photodetachment, ion beam storage, and electron spectrometry. We demonstrate that in the case of OH$^{-}$, the population distribution can be reasonably well described by a Boltzmann distribution. Such method can be useful to estimate the internal population of stored ions in rings or traps where molecular reactions are taking place. The method can be used to provide information on, or comparison with Einstein coefficients, and provide a direct view of the internal dynamics of the relaxation process within a molecular anion. Additionally, such a tool can be very useful for probing the cooling of atomic clusters or polycyclic aromatic hydrocarbons (PAHs), as it provides a direct image of the internal relaxation process as a function of time, and deviation from the simple Boltzmann distribution can be clearly observed.

## Supplementary Information

Supplementary Information.

## Data Availability

The experimental data used and analyses methodologies during the current study are available from the corresponding author on reasonable request.
