# Supplementary material for: Thermometry of stored molecular ion beams
Source: Sci Rep. 2022 Dec 29;12:22518. doi: 10.1038/s41598-022-26797-5 (PMC9800383; doi:10.1038/s41598-022-26797-5)
Supplement: Supplementary file 1 — Supplementary Information. [file 41598_2022_26797_MOESM1_ESM.docx]

Supporting Information (SI)

Thermometry of Stored Molecular Ion Beams
Abhishek Shahi^1^, Deepak Sharma^1^, Sunil Kumar^1^, Saurabh Mishra^1^, Igor Rahinov^2^, Oded Heber^1^, and Daniel Zajfman^1^

^1^Department of Particle Physics and Astrophysics, Weizmann Institute of Science, Rehovot, 7610001, Israel

^2^Department of Natural Sciences, The Open University of Israel, Ra’anana 4310701, Israel

**SI-1: Calibration of VMI setup**

Atomic spectra exhibit pure electronic transitions in general, unlike the molecular spectra that exhibit rotational and vibrational transitions along with electronic transitions. Therefore, a clean spectrum of O¯ is used to calibrate the electron kinetic energy. In the inset figure, VMI data of O¯ is given as done in other work.^1^ In this main figure, a peak corresponds to the ~0.38 eV (obtained from the subtraction of photon energy =1.841 eV and transition energy = 1.461 eV) electron kinetic energy of O¯ reflected as pixel radius #84. Since the radius is proportional to the square of the electron kinetic energy, one can calibrate any radius. We checked this calibration further with both Cs¯ spectrum with three peaks and SIMION simulations.


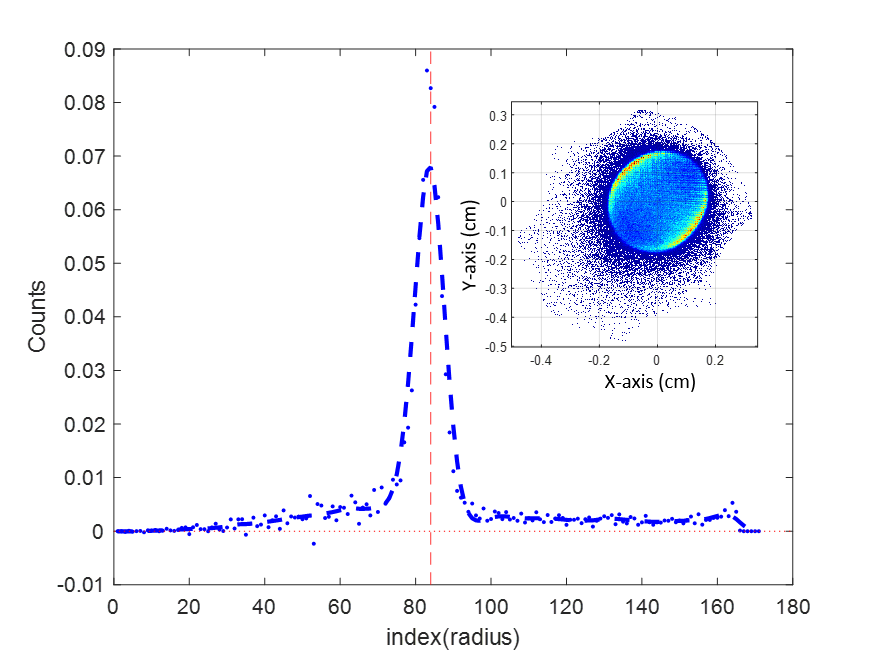


Figure S1: Detector electron energy calibration relating radius to detector position.

VMI spectra of O¯ (insert) raw data.

**SI-2: Background and total counts at the detector**:

We collect the background data while turning off the laser in 10% of the injection along the whole experiment (see Fig. S2 showing the total and the background count at the detector after 81000 injections). Note that while integrating the total counts and background counts, the same types of filters were applied to photoelectron data and background data. The simple signal-to-noise ratio can be calculated as SNR = Total_Counts/(Background_counts*10) = 1232927/(32559*10)  = 3.78

Figure S2: Electron count (in coincidence with neutrals) with laser (black circles) and 10% without laser (green circles).

1. Gale, M. N. *Fine-structure resolved photoelectron spectroscopy of* O*¯ and* OH*¯ via velocity-map imaging*. Master’s thesis, The Australian National University (2009).
